# Removal of pinned scroll waves in cardiac tissues by electric fields in a generic model of three-dimensional excitable media

**DOI:** 10.1038/srep21876

**Published:** 2016-02-24

**Authors:** De-Bei Pan, Xiang Gao, Xia Feng, Jun-Ting Pan, Hong Zhang

**Affiliations:** 1 Zhejiang Institute of Modern Physics and Department of Physics, Zhejiang University, Hangzhou 310027, China; 2 School of Physics and Information Technology, Shaanxi Normal University, Xi’an 710062, China; 3 Faculty of Science, Xi’an Shiyou University, Xi’an 710065, China; 4 Institute of Physical Oceanography and Ocean College, Zhejiang University, Hangzhou 310058, China

## Abstract

Spirals or scroll waves pinned to heterogeneities in cardiac tissues may cause lethal arrhythmias. To unpin these life-threatening spiral waves, methods of wave emission from heterogeneities (WEH) induced by low-voltage pulsed DC electric fields (PDCEFs) and circularly polarized electric fields (CPEFs) have been used in two-dimensional (2D) cardiac tissues. Nevertheless, the unpinning of scroll waves in three-dimensional (3D) cardiac systems is much more difficult than that of spiral waves in 2D cardiac systems, and there are few reports on the removal of pinned scroll waves in 3D cardiac tissues by electric fields. In this article, we investigate in detail the removal of pinned scroll waves in a generic model of 3D excitable media using PDCEF, AC electric field (ACEF) and CPEF, respectively. We find that spherical waves can be induced from the heterogeneities by these electric fields in initially quiescent excitable media. However, only CPEF can induce spherical waves with frequencies higher than that of the pinned scroll wave. Such higher-frequency spherical waves induced by CPEF can be used to drive the pinned scroll wave out of the cardiac systems. We hope this remarkable ability of CPEF can provide a better alternative to terminate arrhythmias caused by pinned scroll waves.

Spiral waves are typical self-organized structures that exist in various systems including chemical media^1^,^2^, aggregations of Dictyostelium discoideum amoebae^3^, and cardiac tissues^4^,^5^. Scroll waves are three-dimensional (3D) extensions of spiral waves, which rotate around one-dimensional singularities known as their filaments^6^,^7^,^8^,^9^,^10^,^11^,^12^. Spiral and scroll waves can also be pinned (anchored) to localized heterogeneities, giving rise to sustained periodic or quasiperiodic activity^13^,^14^,^15^,^16^,^17^,^18^,^19^,^20^,^21^,^22^,^23^,^24^. Pinning is believed to play a crucial role in maintaining abnormal high frequency heart rhythms, including ventricular tachycardia and fibrillation, the main causes of sudden cardiac death^4^,^5^.

Better than the traditional but side-effect therapy of applying a high-voltage electric shock globally, the effect of unpinning by a series of electric pulses, i.e. antitachycardia pacing (ATP)^25^,^26^, is confirmed in both chemical and cardiac systems^27^,^28^,^29^,^30^,^31^. Theoretical analyses are introduced recently to explain its mechanism^32^,^33^. However, the efficiency of a successful unpinning by ATP requires more and better locations of electrodes, and these requirements are not easily accomplished for now.

To overcome the limitations of ATP, a new method using wave emission from heterogeneities (WEH) induced by low-voltage pulsed DC electric fields (PDCEFs) provides a better alternative to terminate arrhythmias^34^,^35^,^36^. This method relies on the effect of the response of cardiac tissue to an external electric field that modifies the membrane potential distribution near the heterogeneities. In hearts, as in many other systems, e.g. nervous network systems^37^,^38^, the complicated nature largely comes from heterogeneities, such as complex anatomical structures, blood vessels, and even tissue damage^36^. If the electric field strength exceeds the excitable threshold, a wave can be nucleated near the heterogeneity. This phenomenon in cardiology is known as virtual electrodes or secondary sources^39^,^40^,^41^,^42^,^43^,^44^,^45^. Using this strategy, unpinning of spiral waves is possible in cardiac tissues by the interaction between the newly generated waves and the anchored spirals^46^,^47^,^48^,^49^,^50^,^51^. Recently, the circularly polarized electric field (CPEF) has shown its unique ability to control spirals and turbulence^52^,^53^,^54^ in chemical media, and has been verified experimentally in the Belousov-Zhabotinsky (BZ) reaction by applying two ACs onto two pairs of field electrodes perpendicular to each other^55^. In ref. 56, we study the mechanism of WEH induced by CPEF in cardiac tissues, and find its ability to successfully unpin anchored spirals is better than that of PDCEF at higher success rate and in larger application scope.

Most of previous works regarding the unpinning only considered two-dimensional (2D) systems, while actually the heart is a 3D object. And the unpinning of scroll waves in 3D systems is much more difficult than that of spiral waves in 2D systems. For 2D systems, after a successful unpinning, the spiral wave will remain free as long as the distance between the obstacle boundary and the spiral tip is larger than the core size of its own^46^,^47^,^48^,^49^,^50^,^51^. For 3D systems, the filament of scroll wave may undergo a deformation in the neighborhood of the obstacle and detach from the obstacle after a suitable electric field is applied, which is similar to the case of spiral wave in 2D systems. However, if we switch off the electric field, the filament would be straightened and re-pinned back to the obstacle again as a result of the positive tension of the filament.

Though unpinning of scroll waves in 3D chemical systems has been investigated^57^,^58^ at present, there are few reports on the removal of pinned scroll waves in 3D cardiac tissues. In this paper, we will focus on removing pinned scroll waves in 3D cardiac tissues using external electric fields in a generic model of excitable media. These electric fields include PDCEF, AC electric field (ACEF) and CPEF, which are shown in Table 1 and Fig. 1. We respectively study the WEH induced by these three forms of external electric fields. We find all of them can induce spherical waves near the obstacles in initially quiescent excitable media. Only CPEF can induce spherical waves whose frequencies are higher than that of the pinned scroll wave. Such higher-frequency spherical waves induced by CPEF may have a unique ability to drive the pinned scroll wave out of the cardiac systems.

## Results

To describe the electrical activities of cardiac tissues, we use the general Barkley model^59^, a simplified mono-domain model:


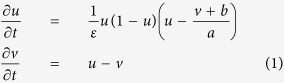


where *u* is the fast variable corresponding to membrane potential, and *v* is a slow variable corresponding to a recovery process. The parameters *a*, *b* and *ε* are fixed at 0.9, 0.08 and 0.02, respectively. When we choose such parameter configurations, the spiral undergoes rigid rotation and the scroll wave filament has positive tension, which corresponds to the “collapsing scroll rings” region in the parameter space of the Barkley model^9^.

**Figure 1 f1:**
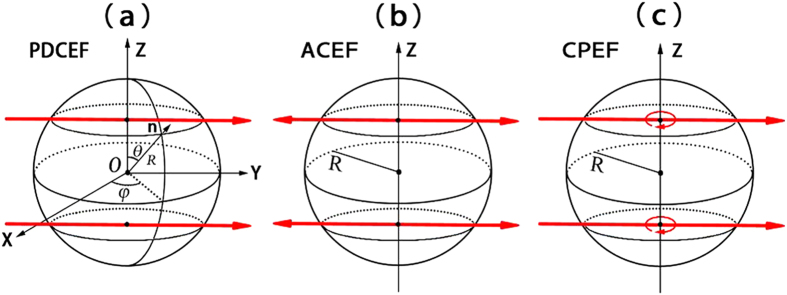
Three forms of external electric fields. The spheres with radius *R* represent the obstacles in cardiac tissues. The red straight arrows represent the directions of PDCEF in (**a**), ACEF in (**b**), and CPEF in (**c**). The red curved arrow in (**c**) means CPEF can rotate horizontally around the z-axis.

In mono-domain models, the general effect of an external electric field on an obstacle can be expressed as a Neumann boundary condition^50^,^51^,^60^:


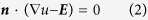


where ***n*** is a local unit vector perpendicular to the obstacle boundary, and ***E*** is the external electric field which represents PDCEF, ACEF or CPEF in this paper. When we apply these three forms of electric fields to a 3D cardiac system, the electric fields will change the membrane potential around the obstacle, respectively (see Fig. 1).

**Figure 2:**
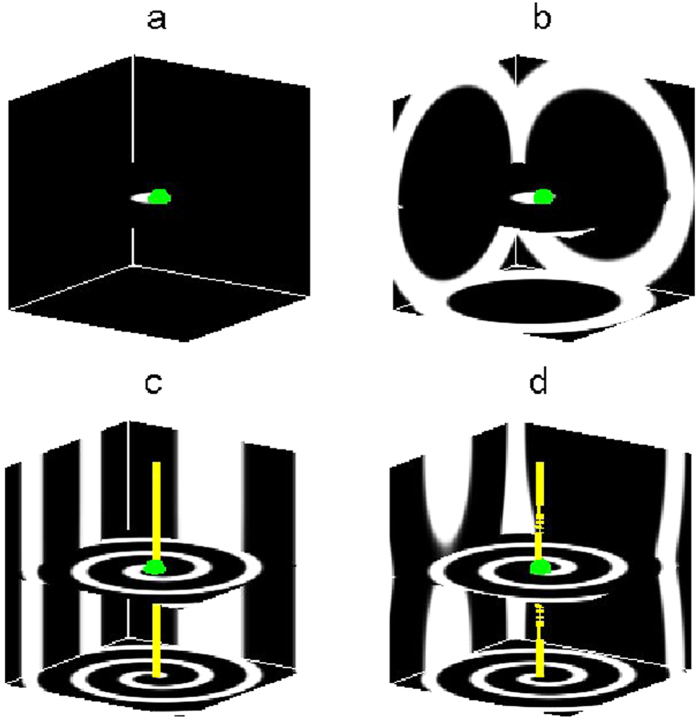
WEH induced by PDCEF. The strength and the frequency of PDCEF are *E*_0_ = 2.0, *ω*_e_ = 3.75, respectively.The central green spheres denote the obstacles with radius 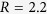. The yellow line, i.e., the filament, represents the rotation center of the scroll wave. **(a,b)** show PDCEF induces spherical waves from the obstacle in a quiescent medium. **(c,d)** show the unsuccessful unpinning of the scroll wave by PDCEF.

Firstly, we study the effect of PDCEF on an initially quiescent excitable medium with an obstacle. By applying PDCEF to the initially quiescent excitable medium, as shown in Fig. 1a, the membrane potential around the obstacle will be redistributed. The de-polarized and hyper-polarized regions are induced on opposite sides of the obstacle respectively, similarly as those in Fig. 1b,d in ref. 56. If the electric field strength is large enough, a wave can be emitted from the de-polarized region and then gradually form a spherical wave near the obstacle, as shown in Fig. 2a. And with the continued effect of the electric field, the spherical wave detaches from the obstacle and propagates outward, then the second spherical wave ensues, and next the third one. Finally, the medium will be dominated by spherical waves, as shown in Fig. 2b.

Now we consider the case that there is a scroll wave initially pinned to the obstacle, as shown in Fig. 2c. By employing the same PDCEF to the system, it is shown that spherical waves cannot be nucleated around the obstacle due to the preceding existence of the pinned scroll wave, as shown in Fig. 2d. That is, the scroll wave is still anchored to the obstacle. Although PDCEF is able to induce spherical waves near the obstacle in a quiescent state, it cannot remove the pinned scroll wave.

**Figure 3:**
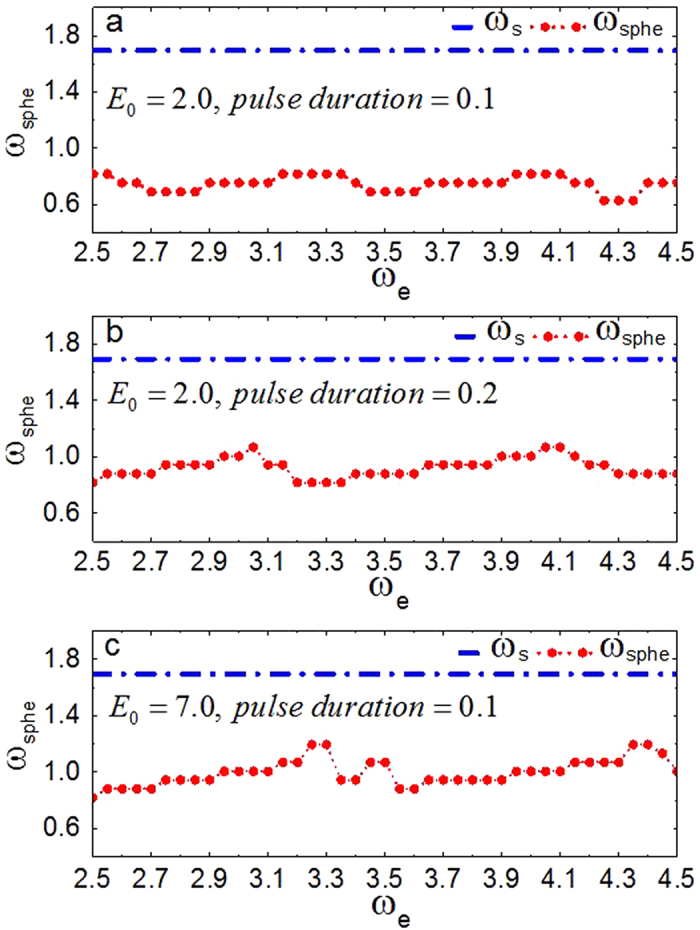
The relationship between the frequencies of the induced spherical wave and of PDCEF. The pulse duration and the strength of PDCEF vary in different subgraphs. (**a**) E0 =2.0 and pulse duration is 0.1, (**b**) E0 = 2.0 and pulse duration is 0.2 and (**c**) E0 =7.0 and pulse duration is 0.1. The dashed line with solid circles represents the frequency of the induced spherical wave 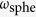 in a quiescent medium. The dash-dotted line represents the frequency of the pinned scroll wave 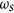.

Considering that PDCEF cannot induce spherical waves with the presence of pinned scroll waves, one may guess that the frequency 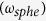 of the induced spherical waves is lower than the frequency 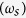 of the pinned scroll wave. So next we will measure the values of 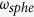 and 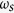, and verify the above suppose. Since the frequency of the generated spherical waves 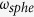 depends on the frequency of electric fields 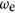, we can obtain a series of spherical waves with different frequencies in a quiescent medium by varying 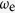. And we find that if 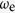 is lower than 2.5, the electric field cannot induce a sound spherical wave initially near the obstacle and these waves may break up subsequently. To study the relation between 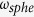 and 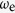, we gradually increase 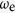 from 2.5 which can be seen as a minimum. Specifically, for a PDCEF with strength 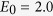 and pulse duration of 0.1 in Fig. 3a, it is shown that no matter how to choose the frequency of PDCEF, it cannot induce spherical waves with frequencies higher than that of the pinned scroll wave. Furthermore, when we increase the pulse duration to 0.2 as shown in Fig. 3b, the results are similar to the case of the pulse duration of 0.1. That is, PDCEF with pulse duration of 0.2 also cannot induce spherical waves whose frequencies are higher than that of the pinned scroll wave. Note that if the pulse duration is 0.05, spherical waves cannot nucleate near the obstacle. On the other hand, changing the strength of PDCEF to 7.0 as shown in Fig. 3c, we cannot get spherical waves with frequencies higher than that of the pinned scroll wave either.

From the above discussion, we can conclude that PDCEF, even with different strength and pulse duration, is not able to induce spherical waves with frequencies higher than that of the pinned scroll wave; thus it cannot remove the pinned scroll wave out of the medium.

**Figure 4:**
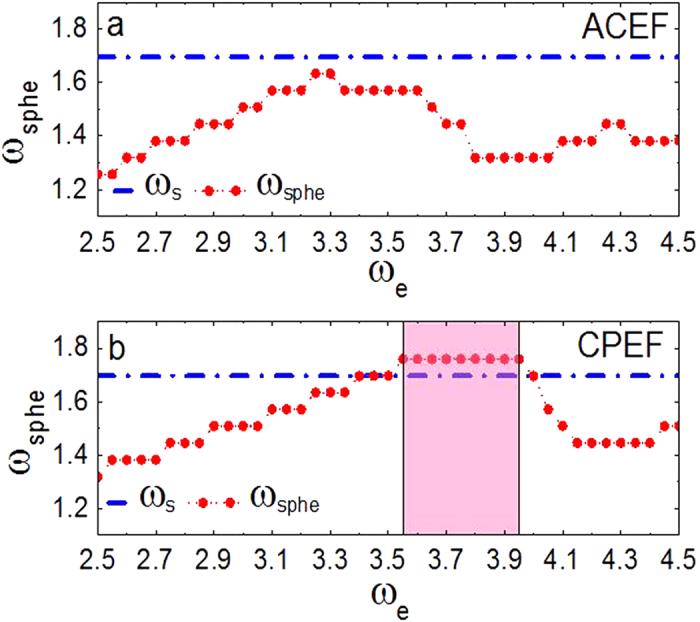
The relationship between the frequencies of spherical waves and of electric fields. The forms of external electric field vary in different subgraphs. (**a**) ACEF and (**b**) CPEF. The strength 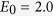. The dashed line with solid circles represents the frequencies of the induced spherical waves 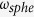 in a quiescent state. The dash-dotted line represents the frequency of the pinned scroll wave 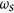.

In the next, we study the medium responding to the other two forms of external electric fields: ACEF and CPEF. For ACEF as shown in Fig. 1b, with the effect of the electric field, the induced de-polarized and hyper-polarized regions are no longer steadily distributed but alternately appeared on opposite sides of the obstacle; thus the waves can be emitted equally on both sides of the obstacle. Although the frequencies of the formed spherical waves induced by ACEF can be higher than those of the spherical waves induced by PDCEF, they are still lower than that of the pinned scroll wave, as shown in Fig. 4a. Even if we increase the strength of ACEF to 4.0 and 7.0, it also cannot induce the spherical waves with frequencies higher than that of the pinned scroll wave. While, if we apply a suitable CPEF, it is capable to obtain higher-frequency spherical waves than that of the pinned scroll wave, as shown in Fig. 4b. This may owe to the unique ability of CPEF: compared to PDCEF and ACEF, the de-polarized and hyper-polarized regions induced by CPEF can rotate around z-axis, as shown in Fig. 1c (similarly as those in Fig. 1a[Fig f1]c in ref. 56). Moreover, we test some other values of parameters *a* and *b* within the “collapsing scroll rings” region in the parameter space of the Barkley model^9^, and find that CPEF can also induce higher-frequency spherical waves than that of the pinned scroll wave.

So far, we have shown that spherical waves can be induced from the obstacle by PDCEF, ACEF and CPEF. But only CPEF can induce spherical waves with frequencies higher than that of the pinned scroll wave. Next, we will give a further discussion on the membrane potential under the influence of these three different electric fields according to Eq. (2). The effect of electric fields on the membrane potential *u* at the boundary of the obstacle can be written as 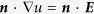. That is, the value of 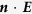 affects the distribution of membrane potential *u* at the boundary of the obstacle. And for any point at the boundary of the spherical obstacle, the local unit vector perpendicular to the obstacle boundary can be expressed as 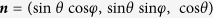 in spherical coordinates (see Fig. 1a). Then we can calculate the value of 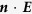, as shown in Table 2. In detail, for PDCEF and ACEF, since they are applied merely in one direction, the extremum of 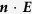 are still relied on *θ* and *φ*. While CPEF can rotate horizontally around the z-axis, so the extremum of 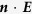 no longer has a dependency on *φ*. And the effect of CPEF on the membrane potential at the boundary of the spherical obstacle is more remarkable than those of PDCEF and ACEF, which to some extent may explain the reason why CPEF can generate spherical waves with frequencies higher than that of the pinned scroll wave.

**Figure 5:**
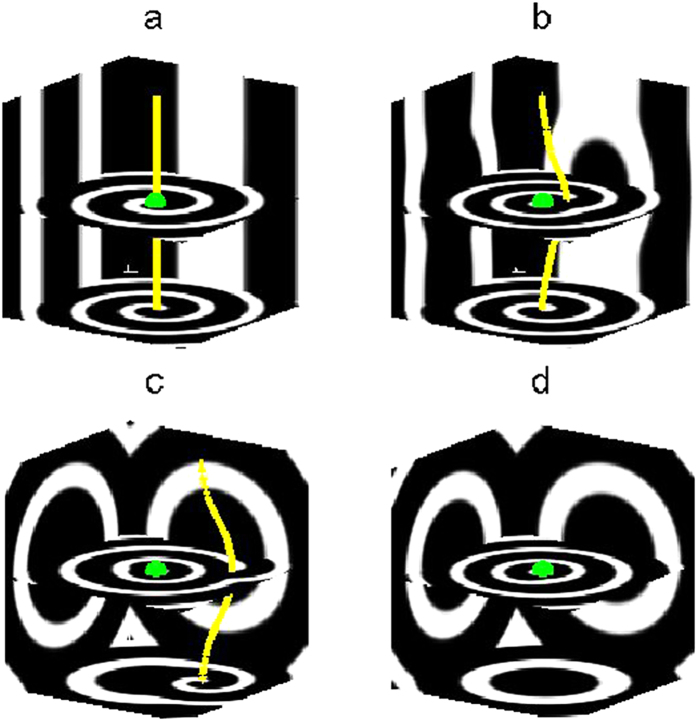
The evolution process for the removal of a pinned scroll wave by CPEF. (**a**), t = 0. (**b**), t = 30. (**c**), t = 160. (**d**), t = 380. The obstacle radius 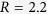, and the frequency of the pinned scroll wave 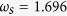. The strength and the frequency of CPEF are 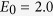, 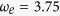, respectively.

In view of the promising findings elucidated above in which the frequency of spherical wave 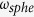 induced by CPEF can be higher than that of the pinned scroll wave 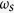, we believe these higher-frequency spherical waves are capable of unpinning the anchored scroll waves and of driving them away out of the medium. We use the same initial state as that in Fig. 2c, and apply a CPEF with 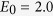, 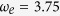 (such a CPEF can induce spherical waves with frequency higher than that of the pinned scroll wave, as shown in Fig. 4b) to the medium. After 30 time units, the filament of scroll wave undergoes a deformation in the neighborhood of the obstacle and then detaches from it (see the yellow line in Fig. 5b). This suggests that the initial unpinning of scroll waves can be obtained due to the interaction between the newly generated waves and the filament. However, if we switch off CPEF at this moment, the filament will be straightened and re-pinned back to the obstacle again as a result of the positive tension of the filament. This is quite different from the unpinning of spirals in 2D systems^46^,^47^,^48^,^49^,^50^,^51^: after a successful unpinning, the spiral wave will remain free as long as the distance between the obstacle boundary and the spiral tip is larger than the core size of its own. Therefore, it is much easier for 2D systems to realize unpinning. As for scroll waves in 3D systems, to achieve definite unpinning, it requires sustainable effects of the CPEF, which can induce higher-frequency spherical waves. As shown in Fig. 5c, scroll wave filament has been driven away far from the obstacle by the higher-frequency outward-propagating spherical waves. Finally, after 380 time units, the scroll wave has been completely swept away out of the medium boundary, and the medium is dominated by spherical waves (see Fig. 5d). When we turn off CPEF at this time, no new spherical waves can be induced near the obstacle afterwards and the remaining spherical waves will move out of the boundary. In the end, the system converts into the quiescent state (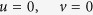). Note that, our further studies show that CPEF with 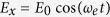, 
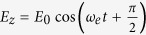
 and with 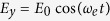, 
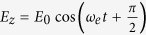
 also can remove the pinned scroll wave out of the medium under the same above strength and frequency of CPEF.

**Figure 6:**
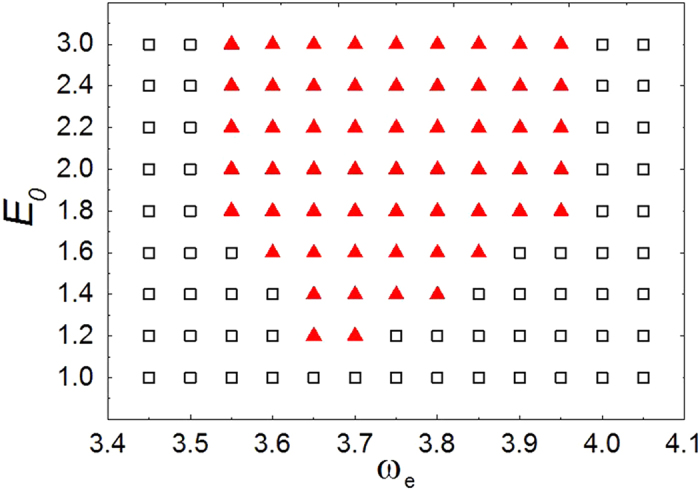
Parameter region of the removal of pinned scroll waves as a function of *E*_*0*_ and *ω*_*e*_. The triangles denote the successful removal by CPEF; the squares the unsuccessful.

Parameter region of the removal of pinned scroll waves as a function of external field amplitude *E*_0_ and frequency 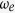 is given in Fig. 6. Specifically, for simplicity we define a box containing the obstacle in its center with a grid size of 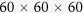. If the scroll wave filament is swept away out of this box by CPEF within 500 time units, we treat it as a successful removal and denote it by a triangle; otherwise, for unsuccessful removal, denote it by a square. From Fig. 6, in particular, we find that when 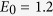, it begins to exist a range of 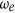 that can lead to successful removal. When we increase the strength *E*_0_ to 1.8, such a “successful removal” range of 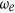 becomes larger as well, which corresponds to the shaded region in Fig. 4b. However, further increasing *E*_0_ makes no contribution to enlarge “successful removal” range any more.

In conclusion, we have demonstrated that, spherical waves can be induced near the obstacle in initially quiescent cardiac tissues by PDCEF, ACEF and CPEF in a generic model of 3D excitable media. The frequencies of all the spherical waves generated by PDCEF are lower than that of the pinned scroll wave. Though the frequencies of the spherical waves induced by ACEF can be higher than those of the spherical waves induced by PDCEF, they are still lower than that of the pinned scroll wave. Only CPEF can induce spherical waves whose frequencies are higher than that of the pinned scroll wave, and these higher-frequency spherical waves can drive the pinned scroll wave out of the medium. The main difference of CPEF from PDCEF and ACEF is the rotation. Because of the rotation of CPEF, the medium will be affected by both de-polarization and hyper-polarization. Note that the model used in this paper is too simple for simulating actual cardiac systems. In order to give some more useful instruction for practical cardiac arrhythmias termination, further investigation taking into account more realistic cardiac activities is needed. On the other hand, CPEF can be easily realized in cardiac tissues by replacing DCs with ACs in the experimental preparation of Fig. 5d in ref. 35, which is similar to the case of the BZ reaction in ref. 55. In this sense, we hope the remarkable ability of CPEF to unpin scroll waves can be verified in cardiac tissues.

## Methods

To add the introduced boundary condition into the spherical boundary of the obstacle in Cartesian coordinates, we adopt the phase field method^50^,^51^,^60^. Considering the effects of an external electric field on the obstacle, Eq. (1) can be adapted as


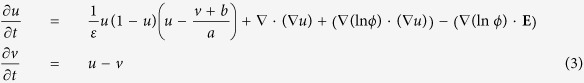


In Cartesian coordinates, Eq. (3) are integrated on a grid size of 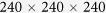 medium with no-flux boundary condition via Euler method, and the central difference method is applied to compute the Laplacian term 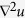 and the gradient terms 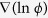,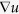. The space and the time steps are 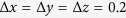 and 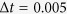, respectively.

## Additional Information

**How to cite this article**: Pan, D.-B. *et al*. Removal of pinned scroll waves in cardiac tissues by electric fields in a generic model of three-dimensional excitable media. *Sci. Rep.* **6**, 21876; doi: 10.1038/srep21876 (2016).

## Figures and Tables

**Table 1:** Three forms of external electric fields.

PDCEF	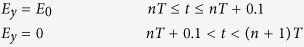 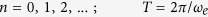
ACEF	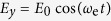
CPEF	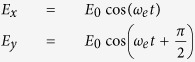

**Table 2:** The effects of electric fields on the membrane potential.

	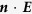	Extremums of 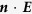
PDCEF	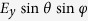	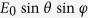
ACEF	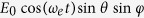	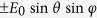
CPEF	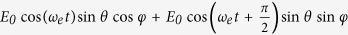	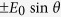
